# Resistance to coronavirus infection in amino peptidase N-deficient pigs

**DOI:** 10.1007/s11248-018-0100-3

**Published:** 2018-10-12

**Authors:** Kristin M. Whitworth, Raymond R. R. Rowland, Vlad Petrovan, Maureen Sheahan, Ada G. Cino-Ozuna, Ying Fang, Richard Hesse, Alan Mileham, Melissa S. Samuel, Kevin D. Wells, Randall S. Prather

**Affiliations:** 1 Division of Animal Science, University of Missouri, Randall Prather, 920 East Campus Drive, Columbia, MO 65211 USA; 2 Department of Diagnostic Medicine and Pathobiology, Kansas State University, Manhattan, KS 66506 USA; 3 Genus plc, Deforest, WI 53532 USA

**Keywords:** Coronavirus, Disease resistance, Viral receptor, CRISPR/Cas9

## Abstract

The alphacoronaviruses, transmissible gastroenteritis virus (TGEV) and Porcine epidemic diarrhea virus (PEDV) are sources of high morbidity and mortality in neonatal pigs, a consequence of dehydration caused by the infection and necrosis of enterocytes. The biological relevance of amino peptidase N (ANPEP) as a putative receptor for TGEV and PEDV in pigs was evaluated by using CRISPR/Cas9 to edit exon 2 of *ANPEP* resulting in a premature stop codon. Knockout pigs possessing the null ANPEP phenotype and age matched wild type pigs were challenged with either PEDV or TGEV. Fecal swabs were collected daily from each animal beginning 1 day prior to challenge with PEDV until the termination of the study. The presence of virus nucleic acid was determined by PCR. *ANPEP* null pigs did not support infection with TGEV, but retained susceptibility to infection with PEDV. Immunohistochemistry confirmed the presence of PEDV reactivity and absence of TGEV reactivity in the enterocytes lining the ileum in *ANPEP* null pigs. The different receptor requirements for TGEV and PEDV have important implications in the development of new genetic tools for the control of enteric disease in pigs.

**Electronic supplementary material:**

The online version of this article (10.1007/s11248-018-0100-3) contains supplementary material, which is available to authorized users.

## Introduction

Respiratory and enteric infections caused by coronaviruses have important impacts on both human and animal health. The infection of immunologically naïve newborn pigs with transmissible gastroenteritis virus (TGEV) or porcine epidemic diarrhea virus (PEDV) results in losses approaching 100% mortality; a consequence of mal-absorptive diarrhea and dehydration caused by the destruction of infected enterocytes (Madson et al. 2016; Saif et al. 2012). Strategies for vaccination of newborn piglets have not been developed and passive immunity is the preferred method of controlling infection (Langel et al. 2016). An outbreak of PEDV on U.S. farms in 2013 resulted in the death of nearly 7 million pigs, an estimated 10% loss in U.S. pig production for that year (Stevenson et al. 2013). Interestingly, TGEV typically causes less destruction in swine herds due to a deletion mutant of TGEV, porcine respiratory corona virus (PRCV) that replicates in the respiratory tract (Kim et al. 2000). Pigs typically recover from PRCV exposure and produce neutralizing antibodies that also neutralize TGEV resulting in a less severe infection in TGEV exposed piglets. In herds that have not been exposed to PRCV, TGEV is similarly lethal to PEDV. Strategies for vaccination of newborn piglets have not been developed and passive immunity is the preferred method of controlling infection (Langel et al. 2016). In older pigs, TGEV and PEDV establish productive, but non-clinical infections (Saif et al. 2012). Recently (2009, 2012 and 2016) three distinct chimeric viruses containing the S gene and 3a sequences of PEDV on a TGEV backbone have been described in Europe. These viruses, named swine enteric coronaviruses, cause clinical signs similar to PEDV but their impact is unknown since standard diagnostic techniques would not distinguish them from their parental viruses (Belsham et al. 2016).

Along with the human, canine and feline coronaviruses, PEDV and TGEV belong to the genus *Alphacoronavirus* in the family *Coronaviridae* (Lin et al. 2015). Coronaviruses are enveloped, single-stranded, positive sense RNA viruses, placed in the order, *Nidovirales*. The characteristic hallmark of nidoviruses is the synthesis of a nested set of subgenomic mRNAs. The unique structural feature of coronaviruses is the “corona” formed by the spike proteins protruding from the surface of the virion. Although the spike protein is the primary receptor binding protein for all coronaviruses, the corresponding cell surface receptors exhibit a wide variation (Li 2015). Delmas et al. (1992) were the first to characterize porcine aminopeptidase N (APN, ANPEP, CD13: although prior convention uses APN as the abbreviation, here we have elected to use the HUGO Gene Nomenclature Committee identifier ANPEP as a candidate receptor for TGEV (HUGO Gene Nomenclature Committee). Porcine ANPEP is a 963 amino acid, type II membrane metallopeptidase responsible for removing N-terminal amino acids from protein substrates during digestion. A variety of cells and tissues have low levels of ANPEP expression, but it is highly expressed on enterocytes. Peptide sequences in the receptor thought to be responsible for binding TGEV include a region in domain VII between amino acids 717 and 813 (Delmas et al. 1994), and the area of overlap between peptides 623–722 and 673–772 (Sun et al. 2012). Additionally, a structural study of the interaction between the spike protein of two coronaviruses and ANPEP implicated residue 736 as a likely binding site for not only TGEV but also porcine respiratory coronavirus; but there is evidence for TGEV binding throughout the protein (Ren et al. 2010). Several studies also identified ANPEP as a putative receptor for PEDV (Kamau et al. 2017; Li et al. 2007; Oh et al. 2003). For example, TGEV and PEDV spike protein N-terminal and C-terminal binding domains (S1-NTD-CTD) recognize porcine ANPEP in dot blot hybridization assays (Li et al. 2016). In addition, PEDV S1-NTD-CTD can bind a second potential receptor, acetylneuraminic acid (Liu et al. 2015). Pseudovirions expressing the PEDV spike protein enter ANPEP-positive porcine cell lines, such as PK-15, and virion entry is blocked with anti-ANPEP antibody. However, the TGEV-permissive porcine cell line, ST, modified to possess a CRIPR/Cas9-mediated knockout of ANPEP was made resistant to TGEV, but retained the capacity to be infected with PEDV (Li et al. 2017). The biological relevance of ANPEP as PEDV receptor is supported by the ability of transgenic mice expressing porcine ANPEP becoming susceptible to infection with the virus (Park et al. 2015). In contrast, two recent papers suggest that ANPEP is not required for PEDV infection (Li et al. 2017; Shirato et al. 2016). Specifically, Shirato et al. 2016 showed that although ANPEP is not the cellular receptor in vitro for PEDV, aminopeptidase activity does promote infectivity.

The purpose of this study was to determine the biological relevance of ANPEP as a virus receptor for PEDV and TGEV by investigating the infection of pigs possessing CRISPR/Cas9-mediated edits in *ANPEP*.

## Results

We first studied the editing ability of six *ANPEP* guide RNAs (gRNAs) in cultured primary porcine fetal fibroblast cells. The six target sequences, all located within exon 2, are listed in Online Resource Table S1. Sequences were designed based on NCBI Reference Sequence, NM_214277.1 and cloned into p330X vector (Addgene). To confirm target specificity for *ANPEP* exon 2, a search of GenBank identified no sequences similar to the gRNAs. The results for 17 gRNA plasmid transfection experiments, presented in Online Resource Table S2, showed that the Guide 2 plasmid possessed the highest editing efficiency followed by Guide 1. However, combining both guides 1 and 2 failed to yield edited cells (see Online Resource Table S2). No *ANPEP*-edits were observed in fibroblasts transfected with the remaining gRNA plasmids.

**Fig. 1:**
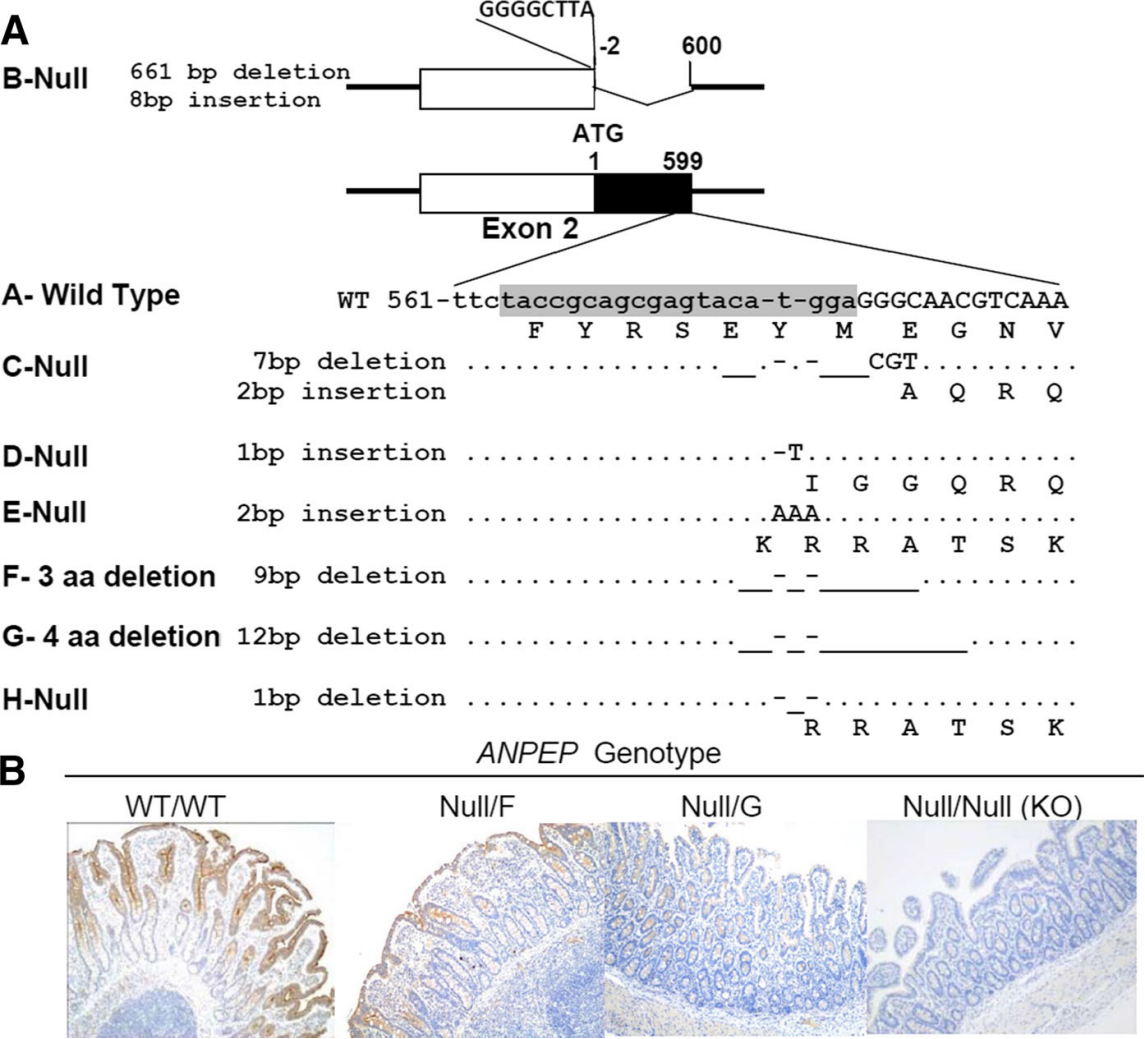
*ANPEP* exon 2 edit alleles used in this study. **A** The CRISPR Guide 2 sequence (highlighted) is located 564 bp downstream of the ATG start codon. The Guide 3 sequence is located 48 bp after the ATG. The left side of the figure shows the allele designation letter followed by a brief description. The amino acids coding for each edit are shown. Key: white area, non-coding region; black area, coding region. The founder animals have the following genotype, 4-2 (D/E), 158-1 (A, D, F, G, H) and 158-9 (B/C). **B** The lower panels show immunoreactivity for ANPEP antigen in ileum sections derived from euthanized PEDV challenged pigs. Ileum sections from *ANPEP* WT pigs showed ANPEP immunoreactivity on the surface of enterocytes lining the intestine. Ileum from pigs possessing either the F or the G allele (9 and 12 bp in frame deletions) also showed immunoreactivity for the ANPEP protein; however, immunoreactivity was visibly weaker in pigs possessing the G allele in which four amino acids were deleted. ANPEP immunoreactivity was absent in pigs possessing two null alleles. Specific genotypes are null/F (B/F), null/G (B/G) and null/null (B/H)

Guide RNAs 2 and 3, along with Cas9 mRNA, were co-injected into the cytoplasm of fertilized oocytes at 14 h after fertilization. Guide 3 was selected because it possessed no editing ability in fibroblasts. It was not clear at the time if guides would have the same editing ability in both fibroblasts and zygotes. Zygotes were cultured for 5 days (morula/blastocyst stage) and transferred to surrogate pigs to gestate (Whitworth et al. 2014). The edited *ANPEP* alleles in offspring piglets were identified based on sequencing PCR products amplified from genomic DNA flanking exon 2. Six embryo transfers resulted in three pregnancies and two litters of viable piglets, which yielded twelve founder animals. Of the 12 founders, nine were edited and of the three founders, a boar and two gilts, were used to create the F1 litters used for the challenge studies (Online Resource Table S3). The exon 2 edits for the three breeding founder pigs are illustrated in Fig. 1A. The *ANPEP*-edited boar, No. 158-9, possessed bi-allelic null edits, consisting of B and C alleles. One *ANPEP*-modified dam, No. 158-1, was a mosaic; possessing a WT (A) allele as well as edits F, G and the null edits, D and H. The F and G alleles possessed 9 and 12 bp deletions, which were predicted to result in the deletion of peptide sequences, 294-Met-Glu-Gly and 294-Met-Glu-Gly-Asp-Val > Ile, respectively. The second dam, No. 4-2, possessed two null alleles, E and D. All frame-shift edits resulted in a premature stop codon. The phenotype of each *ANPEP* edit was confirmed by immunohistochemistry (IHC) for the presence of ANPEP protein in ileum (see Fig. 1B). As expected, all *ANPEP* WT pigs expressed ANPEP on the surface of enterocytes lining the intestine. Phenotypically, pigs possessing either the F or the G allele also showed immunoreactivity for the ANPEP protein; however, immunoreactivity was visibly weaker in pigs possessing the G allele in which four amino acids were deleted. ANPEP immunoreactivity was absent in pigs possessing two null alleles.

**Fig. 2:**
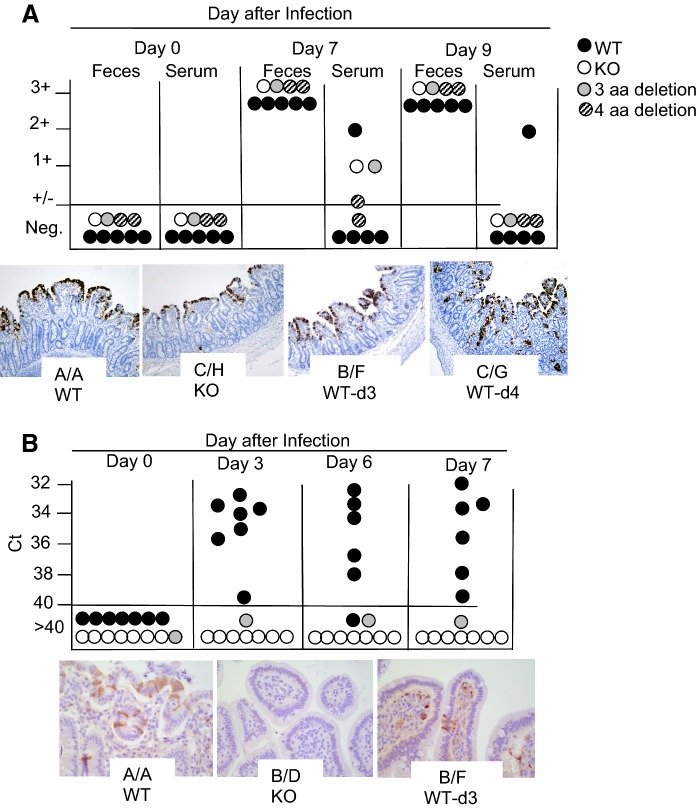
Detection of PEDV and TGEV in WT and *ANPEP*-modified pigs. **A** Nucleic acid in serum and feces of individual pigs was detected by reverse transcriptase (RT)-PCR (*22*). PCR products were separated by electrophoresis on agarose followed by staining with ethidium bromide (EtBr). Results are shown as intensity of EtBr staining; from (3+) for intense staining to (Neg.) for no detectable PCR product. The lower panel shows anti-PEDV antigen IHC of ileum with PEDV anti-spike protein mAb (Cao et al. 2013). **B** RT-PCR detection of TGEV RNA in feces. Results are shown as Ct values. The lower panels show immunoreactivity for TGEV antigen in ileum. The letters under each micrograph identify the *ANPEP* alleles (see Fig. 1) contributing to each phenotype; WT, wild type; KO, ANPEP knockout; WT-d3, three amino acid deletion in ANPEP; WT-d4, four amino acid deletion in ANPEP

Piglets derived from dams No. 158-1 and No. 4-2, artificially inseminated with semen from boar No. 158-9, were utilized for infection with viruses. The first breeding yielded piglets from only No. 158-1. As summarized in Table 1, Litter 121 consisted of eight total piglets, consisting of two pigs possessing the four amino acid deletion, one pig with the three amino acid deletion, and one *ANPEP* KO pig. Five WT pigs from a different litter were included as positive controls for infection. Soon after weaning, all piglets were infected with 10^6^ TCID_50_ of PEDV isolate KS13-09, administered orally. To facilitate continuous exposure to virus, all modified and control pigs were housed in the same pen over the duration of the study. The presence of a productive infection was assessed by the detection of virus in the feces by RT-PCR and by IHC for the presence of viral antigen in ileum. The results, using a standard published assay that provides a good yes or no answer (Niederwerder et al. 2016), presented in Fig. 2A, showed that all pigs were strongly positive for the presence of PEDV nucleic acid in feces, beginning at 7 days after infection. By day 7 post infection, at least one pig of each phenotype was positive by real time PCR for PEDV nucleic acid in serum. IHC confirmed that all pigs possessed PEDV antigen in enterocytes lining the ileum (Fig. 2A). Since all genotypes were infected additional detailed studies were not undertaken. When taken together, these results showed that while ANPEP may play a supporting role for infection (Li et al. 2017; Shirato et al. 2016) the absence of ANPEP did not significantly affect infection of pigs with PEDV.

**Table 1:** Phenotypic and genotypic properties of pigs infected with PEDV

Litter-pig^a^	Phenotype	*ANPEP* Allele		
		Boar	Dam	Sex
121-3	Knockout	C	H	M
121-4	4 aa deletion (d12)	B	G	M
121-5	4 aa deletion (d12)	C	G	F
121-8	3 aa deletion (d9)	B	F	F
145-1	WT	A	A	F
145-2	WT	A	A	F
145-6	WT	A	A	F
145-8	WT	A	A	F
145-9	WT	A	A	F

^a^The *ANPEP*-edited pigs in Litter 121 were derived from the mating of Boar No. 158-9 (Allele 1 = B, Allele 2 = C) with Dam No. 158-1 (Allele 1 = H, Allele 2 = 2 = A, D, F, G, H). The pigs in Litter 145 were normal WT pigs

**Fig. 3:**
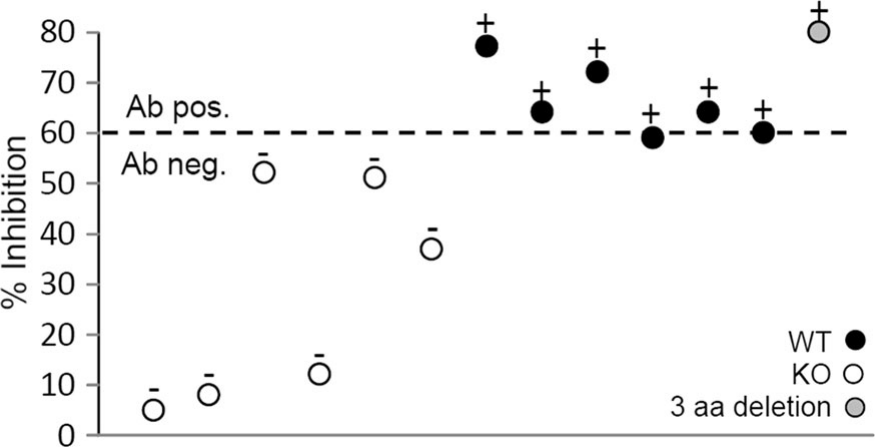
TGEV antibody responses in WT and *ANPEP*—modified pigs. The presence of anti-TGEV antibody in serum at 13 days after infection was measured by blocking ELISA. Each circle represents the result from an individual pig. The horizontal dashed line shows the ELISA cutoff. Pig antibody reactivity against TGEV-infected ST cells, as measured by IFA, is shown as a “+” for positive IFA or “−” for negative IFA

For the second experiment, litters were obtained from both dams. Dam No. 158-1 produced 4 piglets, two of which were used in the study, and dam No. 4-2 produced 13 piglets, six of which were used in the study (summarized in Table 2). Seven WT pigs from a single litter were included as positive controls. Pigs were infected after weaning with the Purdue strain of TGEV by using the same route, dose and housing conditions described above for PEDV. A commercial RT-PCR assay was used to detect the presence of virus in fecal samples. During the first week of infection, all WT pigs were positive for TGEV nucleic acid in feces. Viral nucleic acid was not detected in feces of the single pig possessing the three amino acid deletion in ANPEP or in any of the seven KO pigs (Fig. 2B). The recovery of tissues for the assessment of TGEV antigen IHC was based on the selection of a pig at 3 days after infection, when large quantities of viral nucleic acid were present in feces (Fig. 2B). The WT pig was positive for the presence of TGEV antigen in ileum, while the ANPEP KO pig, removed from the study at the same time, was negative for antigen (Fig. 2B). The intestinal tissue from the pig possessing the three amino acid deletion was process for IHC TGEV antigen at 13 days after infection and showed the presence of antibody for viral antigen in ileum. To confirm the TGEV infection status, serum samples were tested for the presence of TGEV-specific antibody. Both blocking ELISA and indirect immunofluorescence antibody (IFA) method were used to evaluate the presence of TGEV-specific antibodies. The WT and three amino acid deletion pigs were positive for TGEV-specific antibody (Fig. 3); whereas, all ANPEP KO pigs were negative for TGEV antibody. Even though the pig possessing the three amino acid deletion was negative for TGEV nucleic acid in feces, positive TGEV antigen in ileum and a positive antibody response confirmed that this pig was productively infected. Together, these data confirm that the presence of ANPEP is required for TGEV infection of pigs. The successful infection of pigs possessing the minor *ANPEP*-edit that resulted in the deletion of three amino acids in the exon 2 coding region indicates that this peptide sequence is not required for TGEV infection.

**Table 2:** Phenotypic and genotypic properties of pigs infected with TGEV

Litter-pig^a^	Phenotype	*ANPEP* allele		
		Boar	Dam	Sex
20-1	Knockout	B	E	F
20-2	Knockout	C	D	F
20-3	Knockout	C	E	F
20-4	Knockout	C	D	M
20-5	Knockout	B	E	M
20-6	Knockout	C	D	M
127-3	Knockout	C	H	F
127-2	3 aa deletion (d9)	B	F	F
128-1	WT	WT	WT	M
128-3	WT	WT	WT	M
128-4	WT	WT	WT	M
128-5	WT	WT	WT	M
128-6	WT	WT	WT	M
128-7	WT	WT	WT	M
128-8	WT	WT	WT	M

^a^The *ANPEP*-edited pigs in Litter 20 were derived from the mating of Boar No. 158-9 (Allele 1 = B, Allele 2 = C) with Dam No. 4-2 (Allele 1 = D, Allele 2 = E). Two pigs from Litter 127 were the product Boar No. 158-9 mated with No. 158-1 (Allele 1, 2 = A, D, F, G, H). The pigs derived from Litter 128 were normal WT pigs

## Discussion

In vitro models of infection are important tools for the characterization of virus receptors, and for understanding the mechanisms for viral attachment, entry and replication. However, results from in vitro experiments do not necessarily replicate the same findings in the natural host animal, which is composed of a complex set of cells and tissues. For example, mice made transgenic for human ANPEP (hANPEP), a receptor for human coronavirus-229E [HCoV-229E (Yeager et al. 1992)], possess similar levels of expression in the same tissues as mouse ANPEP, including high levels of hANPEP expression in epithelial cells of the intestines (Wentworth et al. 2005). hANPEP-transgenic mice are resistant to infection by intragastric inoculation with HCoV-229E, but cell lines derived from embryos and bone marrow of hANPEP mice support HCoV-229E replication in vitro. These results suggest that other factors besides ANPEP receptor expression on enterocytes are important for infection. In another example, in vitro studies of cells infected with porcine reproductive and respiratory syndrome virus (PRRSV) show that SIGLEC1 on macrophages is required for infection. However, pigs lacking SIGLEC1 support PRRSV infection to the same levels as WT pigs (Prather et al. 2013). Knocking out the expression of another macrophage protein, CD163, conferred complete resistance to PRRSV, demonstrating the requirement for CD163 (Whitworth et al. 2016). Here we confirm that ANPEP is required for the infection of pigs with TGEV, but is not a biologically relevant receptor for PEDV.

The response to a challenge by PEDV showed infectivity in a single *ANPEP* null pig. Since the response is binary, i.e. the animal exhibits viremia or doesn’t exhibit viremia, a single animal can provide the answer to susceptibility to the particular viral stain under investigation. Since our goal was to determine if *ANPEP* null animals are resistant to PEDV a single animal provided the necessary information by showing viral nucleic acid in the feces and PEDV-antigen in the ileum. In contrast, in the second challenge experiment with TGEV we were able to produce more than one litter and had six null animals that did not exhibit a fecal viral load nor TGEV-antigen in the ileum. A single piglet with the 3 bp deletion did not show a fecal viral load, but there was antigen in the ileum. It is possible that the fecal contents affected the PCR assay. For our purposes, we define positive by PCR or histochemically. Since the pigs were cohoused they should all have exposure from both the challenge and the viremia WT animals. When taken together, these results showed that while ANPEP may play a supporting role for infection (Li et al. 2017; Shirato et al. 2016) the absence of ANPEP did not significantly affect infection of pigs with PEDV. Shirato et al. (2016) showed that ANPEP expressing human and porcine cells failed to support PEDV infection, but the same cells were susceptible to infection by TGEV which further validates our model. In the Li et al. (2017), cells were created with a null ANPEP gene via CRISPR/Cas9. The resulting cells were permissive to infectivity by PEDV, but not TGEV. Other coronaviruses may also use ANPEP as an entry mediator. Due to genome similarity porcine respiratory coronavirus (a TGEV mutant) (Zhang et al. 2017) likely uses ANPEP, and other less related viruses such as porcine deltacoronavirus, another swine enteropathogen, may use ANPEP to gain entry into the cell (Li et al. 2018). Clearly additional whole animal studies need to be conducted to better understand the molecules that these viruses use to gain entry. Although PED is exceptionally lethal, neutralizing antibodies against PRCV results in a less severe TGEV infection in exposed piglets. Having a TGEV resistant pig may not be as valuable as a PRRSV or PEDV resistant pig, but this study still provides helpful data on coronavirus entry. Efforts can now be refocused on other mechanisms for PEDV viral entry. ANPEP is a multifunctional protein involved in a variety of physiological and immunological processes (Chen et al. 2012). Pigs possessing a complete knockout of ANPEP appeared normal and indistinguishable from normal littermates, i.e. they grew at normal rates, they reproduced and they did not exhibit any remarkable phenotypes. ANPEP null mice appear to have impaired angiogenesis under stressed conditions (Rangel et al. 2007) and during mammogenesis (Kolb et al. 2013). These phenotypes were neither directly measured nor observed; although sow 4-2 produced sufficient milk for her litter. In ANPEP null mice, there was decrease in thymic T cell numbers, but hematopoietic development, hemostasis, or myeloid cell function were all normal (Winnicka et al. 2010). The founder pigs in this study had no health concerns, but a complete evaluation of their phenotype including macrophage physiology, T-cell changes and mammogenesis was not performed.

Enteric diseases of neonates remain a major source of loss to livestock production, worldwide. These studies demonstrate the feasibility in the use of gene editing to eliminate TGEV, and possibly other viruses, as a source of significant losses to agriculture.

## Methods

All procedures for creating and breeding the pigs were approved by the IACUC at the University of Missouri and viral challenge experiments were approved by the IACUC at Kansas State University.

### Design and cloning of ANPEP guide RNAs (gRNAs)

Guide RNAs were designed to regions within exon 2 of the *ANPEP* gene. Exon two was selected to target for Cas9 cleavage because it includes a large block of coding region and disruption near the start codon would minimize the likelihood that a truncated protein would retain any functional domains. In some predicted transcripts, exon 2 could be placed in part or in whole as the first, second, or third exon. Since the start codon locates to exon 2, all guide RNAs, listed in Online Resource Table S1 and Online Resource Fig. 1, were designed after the start codon so that INDELs would result in a frame shift followed by a premature start codon. The oligonucleotide pairs consisting of primers 1 and 2 were annealed and cloned into the p330X vector, which contains two expression cassettes, a human codon-optimized *S. pyogenes* (hSpy) Cas9 and the chimeric guide RNA. For details see Zhang laboratory protocol (http://www.addgene.org/crispr/zhang/) (Cong et al. 2013; Hsu et al. 2013). Plasmids with the appropriate insert were propagated in Top10 cells (Invitrogen) and plasmid preps were performed with a Qiagen Plasmid Maxi kit (Qiagen). Plasmids were placed at − 20 °C until use for in vitro transcription or for transfection.

### Transfection of fetal fibroblast cells with guide sequence plasmids

Porcine fetuses were collected on day 35 of gestation. One male and one female fetal fibroblast cell line were established from a large white domestic cross. Fetal fibroblasts were collected as described previously with minor modifications (Lai and Prather 2003). Minced tissue from the back of each fetus was digested in 20 mL of digestion media (Dulbecco’s Modified Eagles Medium containing l-glutamine and 1 g/L d-glucose (Cellgro) DMEM supplemented with 200 units/mL collagenase and 25 Kunitz units/mL DNaseI for 5 h at 38.5 °C. After digestion, fetal fibroblast cells were washed and cultured with DMEM supplemented with 15% fetal bovine serum (FBS) and 40 μg/mL gentamicin. After overnight culture, cells were trypsinized and slow frozen to − 80 °C in aliquots in FBS with 10% dimethyl sulfoxide (DMSO) and stored in liquid nitrogen.

Transfection conditions were similar to our previous protocol (Ross et al. 2010; Whitworth et al. 2014). The six *ANPEP* guides were tested in single and in combination with other guides at a concentration of 2 μg of plasmid per transfection. Fetal fibroblast cell lines less than passage four were cultured for 2 days and grown to 75–85% confluency in DMEM supplemented with 15% FBS, 2.5 ng/mL basic fibroblast growth factor (Sigma), 10 mg/mL gentamicin and 25 µg/mL of Fungizone. Fibroblast cells were washed with PBS and trypsinized. After detachment, the cells were rinsed with an electroporation medium (75% cytosalts (120 mM KCl, 0.15 mM CaCl2, 10 mMK2HPO4; pH 7.6, 5 Mm MgCl2)) and 25% OPTI-MEM (Life Technologies, *27*). Cells were counted and pelleted at 600 × g for 5 min and resuspended at a concentration of 1 × 10^6^ cells per ml in electroporation medium. Each electroporation incorporated 200 μL (0.2 × 10^6^ total cells) of cells in 2 mm gap cuvettes with three (1 ms) square-wave pulses administered through a BTX ECM 2001 at 250 V. After the electroporation, cells were resuspended in DMEM medium described above. Colonies were picked on day 14 after transfection. Fetal fibroblasts were plated at 50 cells/plate (Beaton and Wells 2016). Fetal fibroblast colonies were collected by sealing 10 mm autoclaved cloning cylinders around each colony. Colonies were rinsed with PBS and harvested via trypsin; then resuspended in DMEM culture medium. Cells were transferred to a 96-well PCR plate for genotyping.

### Genotyping

PCR was performed using the forward primer 5′ACGCTGTTCCTGAATCT and reverse primer 5′GGGAAAGGGCTGATTGTCTA”, which were incorporated into a standard protocol with LA Taq (Takara). PCR conditions consisted of 96 °C for 2 min followed by 35 cycles of 95 °C for 30 s, 50 °C for 40 s and 72 °C for 1 min and a final extension of 72 °C for 2 min. The 965 bp amplicon product was then separated on a 2.0% agarose gel to examine for the presence of large insertions or deletions combined with Sanger sequencing to determine the exact location of the modification of each allele.

### Preparation of zygotes

Ovaries from pre-pubertal gilts were obtained from an abattoir (Farmland Foods Inc., Milan, MO). Immature oocytes were aspirated from medium size (3–6 mm) follicles using an 18-gauge hypodermic needle attached to a 10 mL syringe. Oocytes with homogenous cytoplasm and intact plasma membrane and surrounding cumulus cells were selected for maturation. Around 50 cumulus oocyte complexes were place in a well containing 500 µL of maturation medium, TCM 199 (Invitrogen) with 3.05 mM glucose, 0.91 mM sodium pyruvate, 0.57 mM cysteine, 10 ng/mL epidermal growth factor (EGF), 0.5 µg/mL luteinizing hormone (LH), 0.5  µg/mL follicle stimulating hormone (FSH), 10 ng/mL gentamicin (APP Pharm), and 0.1% polyvinyl alcohol (PVA) for 42–44 h at 38.5 °C, 5% CO_2_, in humidified air. Following maturation, the surrounding cumulus cells were removed from the oocytes by vortexing for 3 min in the presence of 0.1% hyaluronidase. In vitro matured oocytes were placed in 50 µL droplets of IVF medium (modified Tris-buffered medium (mTBM) with 113.1 mM NaCl, 3 mM KCl, 7.5 mM CaCl_2_, 11 mM glucose, 20 mM Tris, 2 mM caffeine, 5 mM sodium pyruvate, and 2 mg/mL BSA) in groups of 25–30 oocytes. One 100 µL frozen pellet of wild type semen was thawed in 3 mL of DPBS supplemented with 0.1% BSA. Semen was washed in 60% percoll for 20 min at 650 × g and in MTBM for 10 min by centrifugation. The semen pellet was then re-suspended with IVF medium to 0.5 × 10^6^ cells/mL. Fifty microliter of the semen suspension was introduced into the droplets with oocytes. The gametes were co-incubated for 5 h at 38.5 °C in an atmosphere of 5% CO_2_ in air (Abeydeera et al. 1998; Whitworth et al. 2014). After fertilization, the embryos were incubated in PZM3-MU1 (Redel et al. 2015; Yoshioka et al. 2002) at 38.5 °C, 5% CO_2_ in air atmosphere.

### Zygote injection of ANPEP gRNAs

gRNA for zygote injection was prepared as previously described in Whitworth et al. (2017). Guides were cloned into p330X and PCR amplified to provide template DNA to produce guide RNA. A T7 promoter sequence was added upstream of the guide sequence for in vitro transcription. (see Online Resource Table S1). PCR conditions consisted of an initial denaturation of 98 °C for 1 min followed by 35 cycles of 98 °C (10 s), 68 °C (30 s) and 72 °C (30 s). Each PCR amplified template was purified by using a QIAGEN PCR purification kit. Purified amplicons were then used as templates for in vitro transcription using MEGAshortscript (Ambion). RNA quality was visualized on a 2.0% RNA-free agarose gel and concentrations 260:280 ratios were determined via Nanodrop spectrophotometry. Capped and polyadenylated Cas9 mRNA was purchased from Sigma. Guide RNA (20 ng/μl) and Cas9 mRNA (20 ng/μl) were coinjected into the cytoplasm of fertilized oocytes at 14 h post-fertilization by using a FemtoJet microinjector (Eppendorf). Microinjection was performed in manipulation medium (TCM199 with 0.6 mM NaHCO_3_, 2.9 mM Hepes, 30 mM NaCl, 10 ng/mL gentamicin, and 3 mg/mL [BSA]; and osmolarity of 305) on the heated stage of a Nikon inverted microscope (Nikon Corporation). Injected zygotes were then transferred into the PZM3-MU1 with 10 ng/mL PS48 (Stemgent, Inc.) until embryo transfer or allowed to develop to the blastocyst stage for genotype confirmation.

### Embryo transfer

Embryos were cultured for 5 days and then transferred to the oviduct of a gilt on day 4, 5 or 6 of the estrous cycle. All embryos were transported to the surgical site in PZM3-MU1 (Redel et al. 2015) in the presence of 10 ng/mL PS48. Regardless of stage of development, all embryos were surgically transferred into the ampullary-isthmic junction of the oviduct of the recipient gilt (Lee et al. 2013).

### Viruses

PEDV KS13-09 (GenBank No. KJ184549.1) was propagated on Vero76 cells maintained in MEM supplemented with 10% fetal bovine serum (FBS; Sigma), 1% Pen-Strep (Gibco) and 0.25 μg/mL Fungizone. Cells were infected in medium containing 2% Tryptose Phosphate Broth (Sigma), 1 μg/mL L-1-Tosylamide-2-phenylethyl chloromethyl ketone (TPCK)-treated trypsin (Thermo Scientific). For virus titration, Vero76 cells in the 96-well plates were infected with serial 1:10 dilutions of virus in octuplicate at 37 °C with 5% CO_2_. After 3 h, the cell culture medium was replaced with fresh infection medium. At 18 h, the cells were fixed with an acetone:methanol mixture (at 3:2 ratio) for 30 min at 4 °C and reacted with a 1:500 dilution of rabbit polyclonal antibody directed against the PEDV M protein (Genscript). After washing with PBS, FITC conjugated goat-anti-rabbit IgG (Jackson ImmunoResearch) was added as the secondary antibody. Virus concentration was calculated as the TCID_50_/ml using Reed and Muench method (Reed and Muench 1938).

TGEV Purdue strain was cultivated on swine testicular (ST) cells maintained in MEM-FBS, the same as described for PEDV, but without the addition of trypsin. For titration, the virus was serially diluted 1:10 in quadruplicate on confluent ST cells in a 96-well tissue culture plate (BD Falcon). Following 3 days incubation at 37 °C and 5% CO_2_, wells were examined for the presence of cytopathic effect (CPE). The last well showing CPE was used as the titration endpoint and the 50% tissue culture infectious dose (TCID_50_) per ml was calculated according to Reed and Muench (1938).

### Infection of pigs

Experiments involving animals and viruses were performed in accordance with the Federation of Animal Science Societies Guide for the Care and Use of Agricultural Animals in Research and Teaching, the USDA Animal Welfare Act and Animal Welfare Regulations, and were approved by the Kansas State University and University of Missouri institutional animal care and institutional biosafety committees. During the challenge, all infected WT and *ANPEP*-modified pigs were housed together in a single room in the large animal resource center. Genotypes were blinded to the researchers at Kansas State University until after the trial. Therefore, all *ANPEP*-edited pigs received continuous exposure to viruses shed by the infected WT littermates. For infection, pigs received an initial dose of PEDV prepared from a PCR-positive intestinal tissue homogenate from experimentally infected pigs (Niederwerder et al. 2016). Four days later, the pigs were infected a second time with a tissue culture-derived isolate, PEDV KS13-09, which was orally administered as a single 10 mL dose containing 10^6^ TCID_50_ of virus. For TGEV, pigs received the same amount of virus administered orally. Two inoculations were performed to ensure that a productive infection would result.

Fecal swabs were collected daily from each animal beginning 1 day prior to challenge with PEDV until the termination of the study. Each swab was placed in a 15 mL conical tube containing 1 mL of MEM with 1% Pen-Strep and 1% Fungizone. The tube was vortexed briefly to mix the swab contents, aliquoted into 1.5 mL cryovials and then stored at − 80 °C.

### RT-PCR for the detection of viral nucleic acid

Total RNA was extracted from fecal and serum samples using a MagMAX™-96 Total RNA Isolation Kit (Invitrogen™) according to the manufacturer’s instructions on a Thermo Scientific™ KingFisher™ instrument. PEDV RT-PCR was performed using a SuperScript™ III one-step RT-PCR kit with Platinum™ *Taq* DNA polymerase in a total volume of 50 µL. Amplification of PEDV nucleic acid incorporated the forward and reverse primers, 5′ATGGCTTCTGTCAGTTTTCAG and 5′TTAATTTCCTGTGTCGAAGAT, respectively (Niederwerder et al. 2016). PCR was performed as follows: initial reverse transcription at 58 °C for 30 min followed by denaturation at 94 °C for 2 min; and then 40 cycles of 94 °C for 15 s, 48 °C for 30 s, and 68 °C for 90 s. PCR products were visualized on a 1% agarose gel. The results were recorded based on the intensity of ethidium bromide staining.

TGEV nucleic acid was amplified by using a real time procedure (Vemulapalli 2016). Forward primer 5′ TCTGCTGAAGGTGCTATTATATGC, reverse primer 5′CCACAATTTGCCTCTGAATTAGAAG, and 5′ (FAM)YAAGGGCTCACCACCTACTACCACCA(BHQ1) probe were included in the TaqMan^®^ Fast Virus 1-Step Master Mix (Thermo Fisher). RT-PCR included reverse transcription at 50 °C for 30 min, reverse transcription at 95 °C for 15 min followed by 45 cycles of 95 °C for 15 s, 56 °C for 30 s and 72 °C for 15 s. PCR was performed on a CFX-96 real-time PCR system (Bio-Rad) in a 96-well format and the result for each sample reported as a Ct value.

### Immunohistochemistry (IHC) for detection of viral antigen in tissues

Upon collection, intestinal tissues were immediately placed in 10% buffered formalin. After processing, the paraffin-embedded sections were mounted on slides. Sections were dewaxed with Leica Bond Dewax Solution and antigen retrieval performed using Bond Epitope Retrieval Solution 1 (Leica) for 20 min at 100 °C. Slides were incubated with 3% hydrogen peroxide for 5 min at room temperature and visualized by using an automated procedure on a NexES IHC Staining Module (Ventana Medical). A rabbit anti-CD13 (ANPEP, APN) polyclonal antibody (Abcam) prepared against a peptide covering amino acids 400–500 of human CD13 was used for the detection of ANPEP antigen. The antibody was diluted 1:3200 in Bond Primary Antibody Diluent (Leica) and incubated on slides for 15 min at room temperature. Slides were washed and bound antibody detected with anti-Rabbit IgG HRP, which was included in the kit. HRP activity was visualized with DAB and slides counter stained with hematoxylin. The PEDV and TGEV IHC were performed by using similar methods as a routine diagnostic test by the Kansas State University and University of Missouri veterinary diagnostic laboratories.

### Detection of TGEV-specific antibody in serum

Blocking ELISA and indirect immunofluorescence antibody (IFA) were used to detect TGEV-specific antibodies in serum. For IFA, confluent ST cells on 96 well plates were infected with 200 TCID_50_/ml of TGEV Purdue. After 3 days incubation at 37 °C and 5% CO_2_, cells were fixed with 80% acetone. Serum from each pig was serially diluted in PBS with 5% goat serum (PBS-GS). A serum sample obtained from each pig prior to infection served as a negative control. After incubation for 1 h at 37 °C, plates were washed and secondary antibody added to each well. Alexa-Fluor-488 AffiPure goat anti-swine IgG (Cat# 114-545-003, Jackson ImmunoResearch) was diluted 1:400 dilution in PBS-GS. Plates were incubated for 1 h at 37 °C, washed with PBS, and viewed under a fluorescence microscope. The presence of fluorescence at a serum dilution of 1:5 or greater was considered positive for the presence of antibody. Antibody blocking assays were performed using a kit from Sanova (Svanovir TGEV/PRCV). Assays were performed according to the kit instructions and results reported as percent inhibition of binding of labeled TGEV-specific antibody.

## Electronic supplementary material

Below is the link to the electronic supplementary material.

Supplementary material 1 (DOC 377 kb)
