# Supplementary material for: Resistance to coronavirus infection in amino peptidase N-deficient pigs
Source: Transgenic Res. 2018 Oct 12;28(1):21–32. doi: 10.1007/s11248-018-0100-3 (PMC6353812; doi:10.1007/s11248-018-0100-3)

**List of Supplemental Resources**

**Table S1**

**Table S2**

**Table S3**

| **Table S1. Oligonucleotides used for cloning CRISPR gRNA sequence into plasmid p330X and in vitro transcription (IVT)*.** | |
| --- | --- |
| *ANPEP* Guide | Sequence (5'-3') |
| Guide 1 | CTTCTACCGCAGCGAGTACA(TGG) |
| Primer 1 For | CACCGCTTCTACCGCAGCGAGTACA |
| Primer 2 Rev | AAACTGTACTCGCTGCGGTAGAAGC |
| IVT For | TTAATACGACTCACTATAGGCTTCTACCGCAGCGAGTACA |
| Guide 2 | TACCGCAGCGAGTACATGGA(GGG) |
| Primer 1 For | CACCGTACCGCAGCGAGTACATGGA |
| Primer 2 Rev | AAACTCCATGTACTCGCTGCGGTAC |
| IVT For | TTAATACGACTCACTATAGGTACCGCAGCGAGTACATGGA |
| Guide 3 | CCTCCTCGGCGTGGCGGCCG(TGG) |
| Primer 1 For | CACCGCCTCCTCGGCGTGGCGGCCG |
| Primer 2 Rev | AAACCGGCCGCCACGCCGAGGAGGC |
| IVT For | TTAATACGACTCACTATAGGCCTCCTCGGCGTGGCGGCCG |
| Guide 4 | CACCATCATCGCTCTGTCTG(TGG) |
| Primer 1 For | CACCGCACCATCATCGCTCTGTCTG |
| Primer 2 Rev | AAACCAGACAGAGCGATGATGGTGC |
| IVT For | TTAATACGACTCACTATAGGCACCATCATCGCTCTGTCTG |
| Guide 5 | TACCTCACTCCCAACGCGGA(TGG) |
| Primer 1 For | CACCGTACCTCACTCCCAACGCGGA |
| Primer 2 Rev | AAACTCCGCGTTGGGAGTGAGGTAC |
| IVT For | TTAATACGACTCACTATAGGCACCATCATCGCTCTGTCTG |
| Guide 6 | AGCTCAACTACACCACCCAG(GGG) |
| Primer 1 For | CACCGAGCTCAACTACACCACCCAG |
| Primer 2 Rev | AAACCTGGGTGGTGTAGTTGAGCTC |
| IVT For | TTAATACGACTCACTATAGGAGCTCAACTACACCACCCAG |
| gRNA Rev | AAA AGC ACC GAC TCG GTG CC |
| *1 Primer 1 and primer 2 of each guide sequence were annealed and cloned into the p330X vector. The PAM sequence in the resulting guide is shown in parenthesis. IVT For and gRNA Rev primers were used to amplify the specific guide sequence used as a template for *in vitro* RNA synthesis. | |

| **Table S2: Summary of fetal fibroblast transfection with *ANPEP* gRNA plasmids.** | | | | | | |
| --- | --- | --- | --- | --- | --- | --- |
| **No.** | **gRNA target** | **Number of Colonies** | **Number of Plates** | **Colonies**  **Per plate** | **Number of Edited Colonies** | **%** |
| 1 | ANPEP 1 | 42 | 17 | 2.47 | 3 | 7.1 |
| 2 | ANPEP 2 | 31 | 12 | 2.58 | 1 | 3.2 |
| 3 | ANPEP 1+2 | 23 | 19 | 1.21 | 0 | 0.0 |
| 4 | ANPEP 1 | 27 | 10 | 2.70 | 1 | 3.7 |
| 5 | ANPEP 2 | 30 | 10 | 3.00 | 7 | 23.3 |
| 6 | ANPEP 1+2 | 14 | 10 | 1.40 | 0 | 0.0 |
| 7 | ANPEP 1 | 46 | 10 | 4.60 | 0 | 0.0 |
| 8 | ANPEP 2 | 36 | 10 | 3.60 | 0 | 0.0 |
| 9 | ANPEP 3 | 40 | 10 | 4.00 | 0 | 0.0 |
| 10 | ANPEP 4 | 35 | 10 | 3.50 | 0 | 0.0 |
| 11 | ANPEP 1 | 41 | 10 | 4.10 | 1 | 2.4 |
| 12 | ANPEP 2 | 21 | 10 | 2.10 | 3 | 14.3 |
| 13 | ANPEP 1+2 | 34 | 10 | 3.40 | 0 | 0.0 |
| 14 | ANPEP 3 | 28 | 10 | 2.80 | 0 | 0.0 |
| 15 | ANPEP 4 | 33 | 10 | 3.30 | 0 | 0.0 |
| 16 | ANPEP 5 | 35 | 10 | 3.50 | 1 | 2.9 |
| 17 | ANPEP 6 | 24 | 10 | 2.40 | 1 | 4.2 |

| **Table S3. Embryo transfer results from *in vitro* fertilization-derived zygotes directly injected with of *ANPEP* gRNAs.** | | |
| --- | --- | --- |
| **Pig ID** | **# Embryos Transferred** | **Outcome and Results for PCR Sequencing** |
| O345 | 52 morula/blastocyst stage | Collected fetuses at day 35 |
|  |  | One fetus-1 bp biallelic deletion, uncharacterized allele |
|  |  | One fetus-monoallelic, 2 bp deletion, WT |
|  |  | One fetus-mosaic, 2 bp deletion, 9 bp deletion, WT |
|  |  | One fetus- monoallelic, 9 bp deletion, WT |
|  |  | Two WT fetuses |
| O432 | 68 morula/blastocyst stage | No pregnancy |
| O448 | 60 morula/blastocyst stage | Farrowed |
|  |  | Two pigs-monoallelic 9 bp deletion, WT |
|  |  | One pig- biallelic, 1 bp insertion, 2 bp insertion*^1 (4-2,D/E)^ |
|  |  | WT |
| O606 | 63 morula/blastocyst stage | No Pregnancy |
| O642*^2^ | 60 morula/blastocyst stage | Farrowed |
|  |  | One pig- mosaic, 1 bp deletion, 1 bp insertion, 12 bp insertion, 9 bp deletion, WT *^3 (158-1, A, D, F, G, H)^ |
|  |  | One pig-mosaic, 1 bp insertion, 1 bp deletion, 25  bp deletion, 2 bp mismatch |
|  |  | One pig-biallelic, 8 bp deletion, 2 bp mismatch |
|  |  | One pig-biallelic, 1 bp insertion, 2 bp insertion |
|  |  | One pig-biallelic, 9 bp deletion, 1 bp mismatch |
|  |  | One pig-biallelic, 1 bp insertion, 2 bp insertion |
|  |  | One pig-biallelic, 661 bp deletion+8 bp insertion,  7 bp deletion+3 bp addition*^4 (158-9, B, C)^ |
|  |  | Two pigs-WT |
| O533*^2^ | 70 morula/blastocyst stage | No pregnancy |
| *^1^ Founder female for table 2  *^2^ Oocytes were cultured in FLI-treated medium (Yuan et al. 2017)  *^3^ Founder females for litters in tables 1 and 2  *^4^ Founder male for litters in tables 1 and 2 | | |

**References:**

Yuan Y, Spate LD, Redel BK, Tian Y, Zhou J, Prather RS, Roberts RM (2017) Quadrupling efficiency in production of genetically modified pigs through improved oocyte maturation Proc Natl Acad Sci U S A 114:E5796-E5804 doi:10.1073/pnas.1703998114

Figure S1: CRISPR guide RNAs were designed along *ANPEP* exon 2. The sequence of each guide is underlined and the corresponding PAM sequence is in bold font.


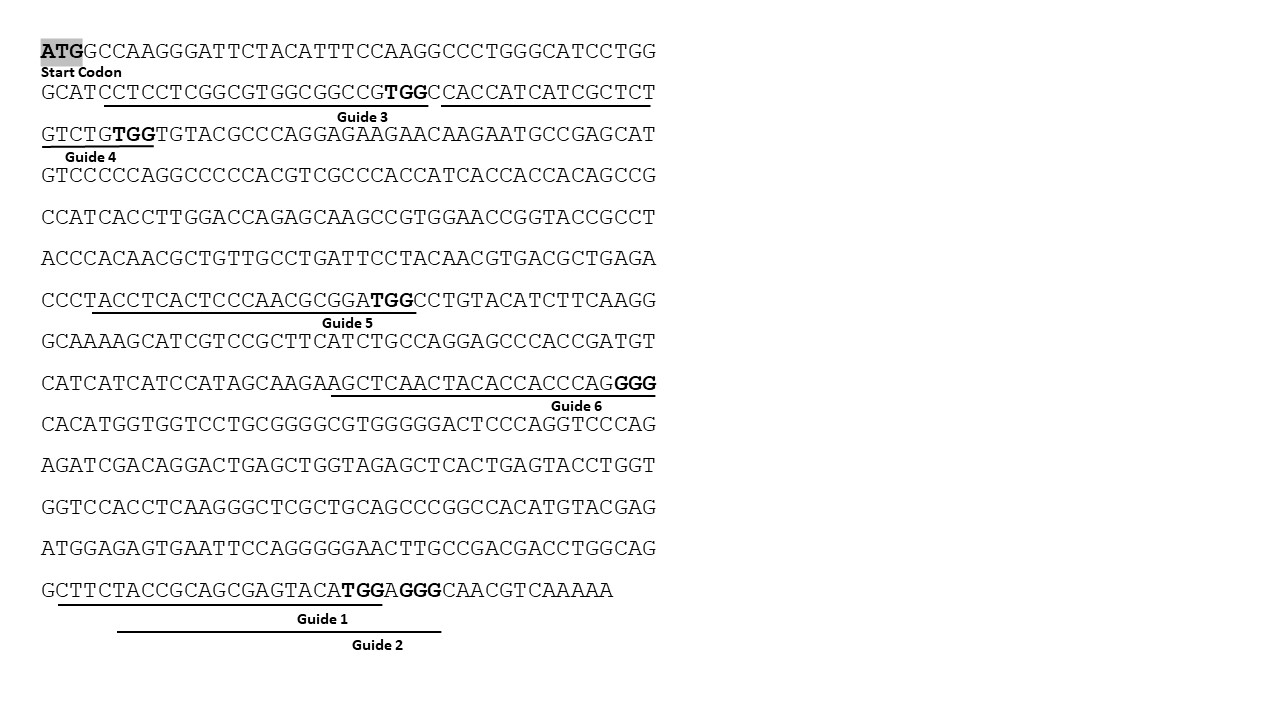

Supplement: Supplementary file 1 — Supplementary material 1 (DOC 377 kb) [file 11248_2018_100_MOESM1_ESM.doc]
